# Influenza and Local Immunity

**Published:** 1907-01-19

**Authors:** Charles J. Whitby

**Affiliations:** Bloomfield Avenue, Bath


### Editor's Letter-Box.

Our Correspondents are reminded that prolixity is a great bar tc publication, and that brevity of style and conciseness of statement greatly facilitate early insertion.]

### INFLUENZA AND LOCAL IMMUNITY.

*To the Editor of* The Hospital.

Sir,—Referring to my article on "Influenza and Local Immunity" in The Hospital for December 22 last, will you permit me to state publicly that I do not hold any such appointment as resident physician to the Smedley Memorial Hospital, Bath? The addition of this erroneous title to my signature may have been due to the fact that I was at one time consulting physician to the Smedley Memorial Hospital, Matlock. Yours, etc.,

Charles J. Whitby, M.D. Camb.

Bloomfield Avenue, Bath.

